# A Novel Interleukin 33/ST2 Signaling Regulates Inflammatory Response in Human Corneal Epithelium

**DOI:** 10.1371/journal.pone.0060963

**Published:** 2013-04-09

**Authors:** Jing Lin, Lili Zhang, Guiqiu Zhao, Zhitao Su, Ruzhi Deng, Stephen C. Pflugfelder, De-Quan Li

**Affiliations:** 1 Department of Ophthalmology, the Affiliated Hospital of Qingdao University Medical College, Qingdao, China; 2 Ocular Surface Center, Cullen Eye Institute, Department of Ophthalmology, Baylor College of Medicine, Houston, Texas, United States of America; 3 School of Optometry and Ophthalmology, Wenzhou Medical College, Wenzhou, China; University of São Paulo, Brazil

## Abstract

Interleukin (IL) 33, a member of IL-1 cytokine family, is well known to promote Th2 type immune responses by signaling through its receptor ST2. However, it is not clear whether ST2 is expressed by mucosal epithelium, and how it responds to IL-33 to induce inflammatory mediators. This study was to identify the presence and function of ST2 and explore the role of IL-33/ST2 signaling in regulating the inflammatory cytokine production in corneal epithelial cells. Human corneal tissues and cultured primary human corneal epithelial cells (HCECs) were treated with IL-33 in different concentrations without or with different inhibitors to evaluate the expression, location and signaling pathways of ST2 in regulating production of inflammatory cytokine and chemokine. The mRNA expression was determined by reverse transcription and real time PCR, and protein production was measured by enzyme-linked immunosorbent assay (ELISA), immunohistochemical and immunofluorescent staining. ST2 mRNA and protein were detected in donor corneal epithelium and cultured HCECs, and ST2 signal was enhanced by exposure to IL-33. IL-33 significantly stimulated the production of inflammatory cytokines (TNF-α, IL-1β and IL-6) and chemokine IL-8 by HCECs at both mRNA and protein levels. The stimulated production of inflammatory mediators by IL-33 was blocked by ST2 antibody or soluble ST2 protein. Interestingly, the IκB-α inhibitor BAY11-7082 or NF-κB activation inhibitor quinazoline blocked NF-κB p65 protein phosphorylation and nuclear translocation, and also suppressed the production of these inflammatory cytokines and chemokine induced by IL-33. These findings demonstrate that ST2 is present in human corneal epithelial cells, and IL-33/ST2 signaling plays an important role in regulating IL-33 induced inflammatory responses in ocular surface.

## Introduction

Interleukin (IL) 33, a new member of IL-1 cytokine family, has been well characterized as a potent inducer of T helper (Th) 2 immune responses [1]. IL-33 potently induces the production of Th2-associated cytokines IL-4, IL-5 and IL-13 released from polarized Th2 cells [1], mast cells [2], [3] and basophils [4]. IL-33 appears to be a cytokine with dual function, acting as a proinflammatory cytokine and as an intracellular nuclear factor with transcriptional regulatory properties [5]. IL-33 is expressed in various types of cells, including epithelial cells, endothelial cells, fibroblasts and smooth muscle cells [6]–[8]. Epithelial-derived IL-33 is critical regulators of innate and adaptive immune responses associated with Th2 cytokine-mediated allergic inflammation [9], [10]. In addition to allergic and autoimmune effects, IL-33 also represents an important mediator of mucosal epithelial restoration and repair [11]. However, the inflammatory response in mucosal epithelium induced by IL-33 remains to be elucidated.

Originally identified 23 years ago as a serum-inducible secreted protein in murine growth-stimulated fibroblast [12], [13], ST2 in its transmembrane form is expressed primarily on mast cells and on Th2 cells and is linked to important Th2 effector functions [14]. As one of IL-1 receptor family members, ST2 had eluded ligand identification until 2005 when Schmitz et al. first identified the orphan receptor ST2 as a receptor for IL-33 [1]. The ST2 gene is now known to encode at least 3 isoforms of ST2 proteins by alternative splicing: a trans-membrane receptor ST2L; a secreted soluble ST2 form which can serve as a decoy receptor for IL-33; and ST2V, a variant form present mainly in the gut of humans [15]. ST2L (also known as T1, IL-1RL1, and DER4) is a member of the TLR/IL1R superfamily, which shares a common structure with an extracellular domain of three linked immunoglobulin-like motifs, a transmembrane segment and a cytoplasmic Toll-interleukin-1 receptor (TIR) domain.

After identification of IL-33 as a novel ligand of ST2, more investigators reported the expression and function of IL-33/ST2 signaling in various types of cells. ST2/IL-33 overstimulation has been implicated in allergic and autoimmune diseases such as arthritis [16], airway hyperactivity and asthma [17], [18], demonstrating an important role of ST2 in the development of Th2-dominant inflammatory pathologies. However, the expression and function of ST2 in epithelium, especially mucosal tissues such as corneal epithelium, are not clear, although a few studies showed ST2 significantly increased inflammatory cytokines in retinal pigment epithelium (RPE) cells very recently [19]. In this study we demonstrated, for the first time, that ST2 is present in human corneal epithelium, and the IL-33 stimulated the expression and production of pro-inflammatory cytokine and chemokine via ST2 mediated NF-κB signaling pathways in human corneal epithelial cells.

## Results

### ST2 was Detected in Human Corneal Epithelium ex vivo and its Primary Cultures in vitro

**Figure 1:**
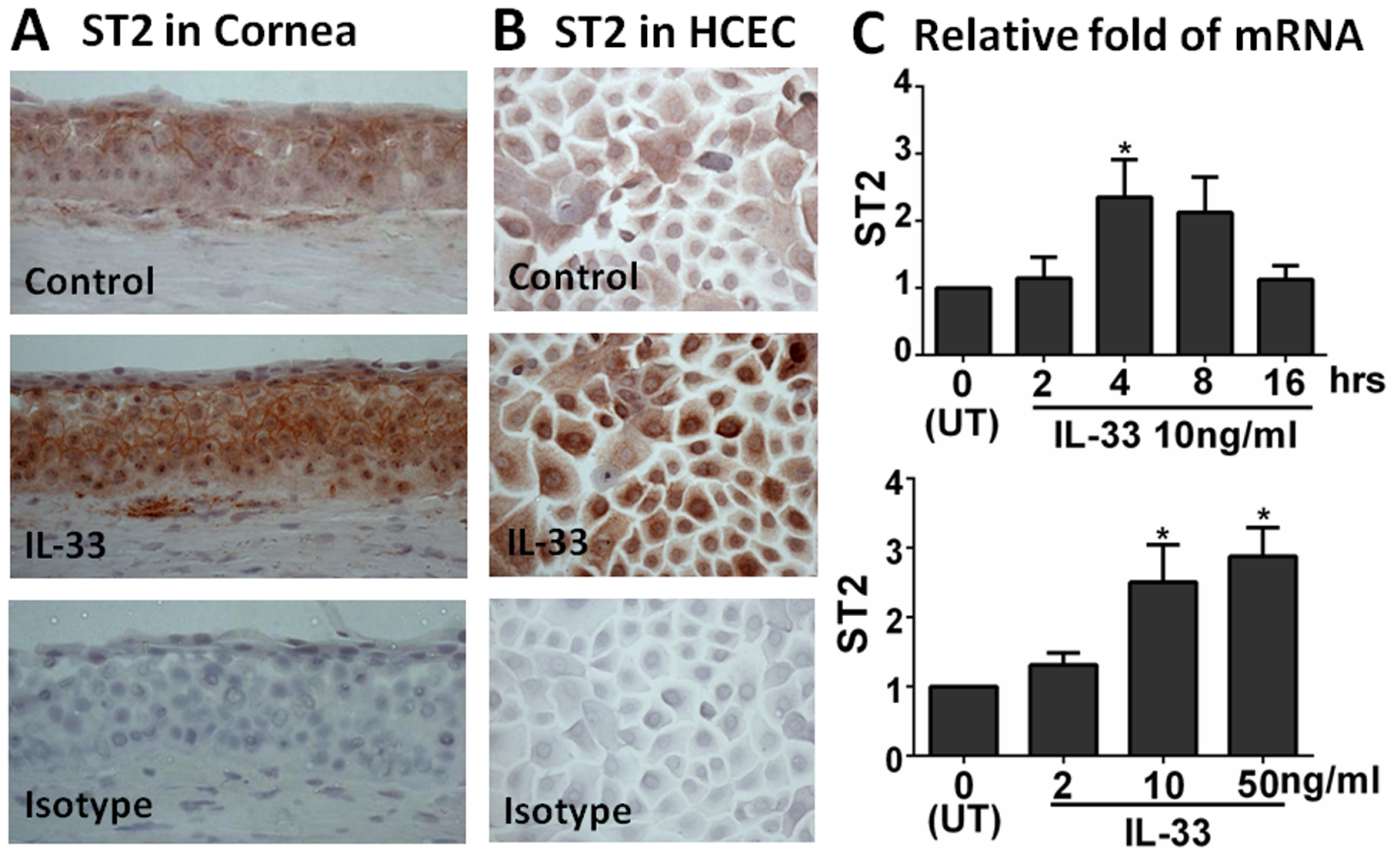
ST2 was expressed by human corneal epithelium. **A.** Representative images showing ST2 localization in ex vivo donor corneal tissues without or with exposure to IL-33 (10 ng/ml) by immunohistochemical staining with isotype IgG as a negative control. **B.** Immunohistochemical images showing ST2 protein in primary HCECs without or with exposure to IL-33, with isotype IgG as a negative control. Magnification 400×. **C.** The mRNA expression of ST2 by HCECs exposed to IL-33 in time course and dose response.

To investigate the cellular location and stimulation of ST2 protein ex vivo, fresh donor corneal tissues were incubated with IL-33 (10 ng/ml) for 48 h, followed by Immunohistochemical staining. As shown in Fig. 1A, the ST2 protein mainly located in the cell membrane and cytoplasm in the superficial epithelial layers of normal donor corneas. Stronger immunoreactivity throughout multiple layers of corneal epithelium was observed in the tissues exposed to IL-33 for 48 h. In primary human corneal epithelial cells (HCECs) cultured from explants of donor corneal limbal tissues, we observed that immunohistochemical staining of ST2 was located mainly in the cytoplasm, and stronger cytoplasmic and more nuclear staining was observed in cultures exposed to IL-33 (Fig. 1B).

The presence of ST2 in human corneal epithelium was further confirmed at mRNA transcriptional levels as evaluated by reverse transcription and quantitative real-time PCR (RT-qPCR). As shown in Fig. 1C, the mRNA of ST2 was expressed in untreated primary HCECs, and it was stimulated by 2- to 3-fold with a peak level at 4 hrs after exposure to IL-33 (10 ng/ml). The stimulation of ST2 by IL-33 appeared in a dose-dependent manner when tested with 2, 10, and 50 ng/ml. The findings indicate the presence of ST2 in human corneal epithelium.

### IL-33 Stimulated Expression and Production of Pro-inflammatory Mediators by HCECs

**Figure 2:**
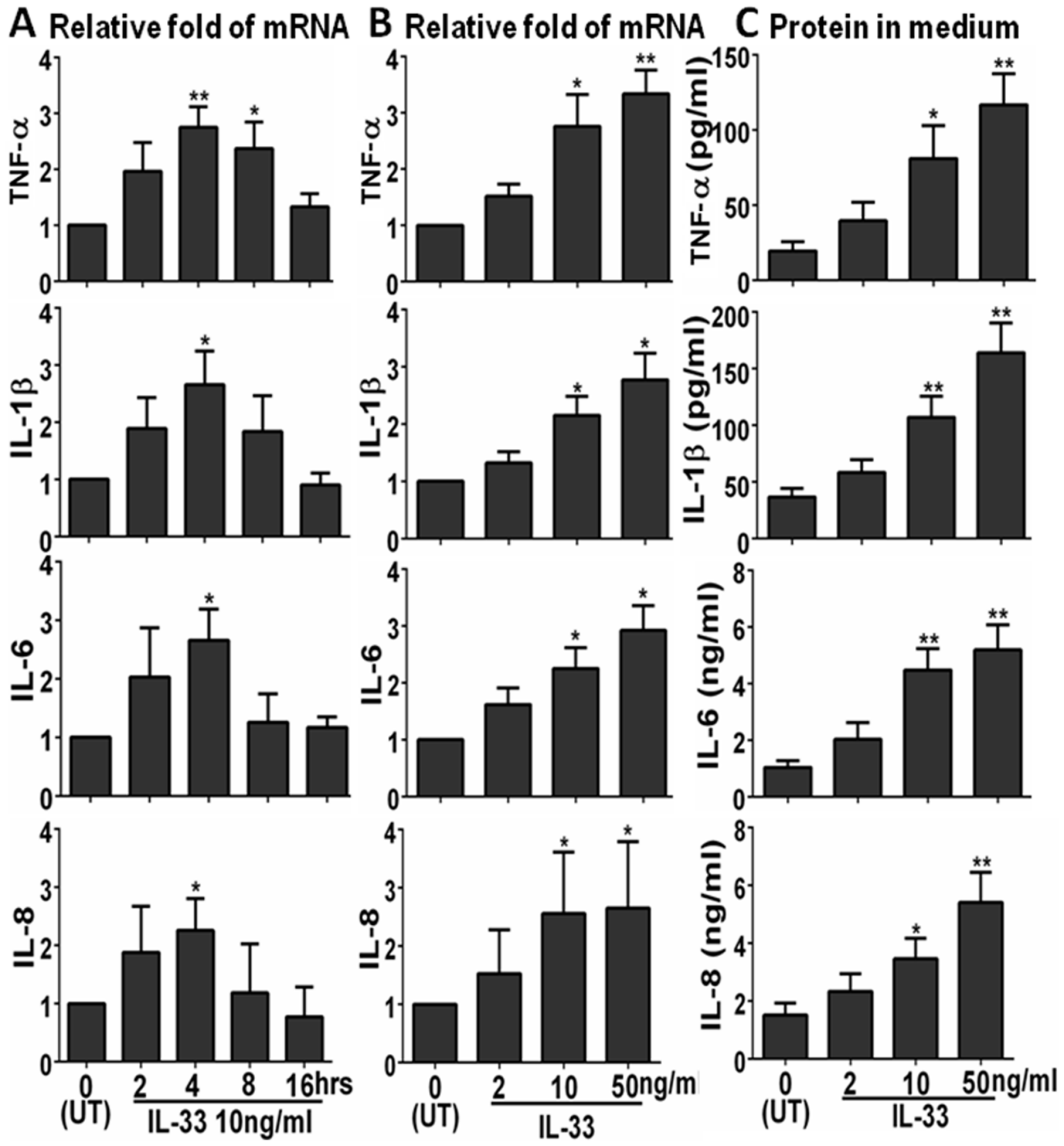
IL-33 induced inflammatory mediators in HCECs with time course and dose response. The expression of inflammatory cytokines (TNF-α, IL-1β and IL-6) and chemokine IL-8 were measured by RT-qPCR for mRNA (**A** & **B**) and by ELISA for protein levels in culture supernatants (**C**). Results shown are mean ± SD of four independent experiments. *p<0.05; **p<0.01, n = 4.

To explore the role of IL-33 in inflammatory response by corneal epithelium, we evaluated the mRNA expression and protein production of pro-inflammatory cytokines (TNF-α, IL-1β, and IL-6) and chemokine IL-8 in primary HCECs by RT-qPCR and ELISA, respectively. With the untreated primary HCECs as controls, the mRNA expression of TNF-α, IL-1β, IL-6 and IL-8 was significantly induced (up to 3-fold) in cells exposed to IL-33 (10 ng/ml) for 4 hrs (Fig. 2A). The stimulatory effects on these inflammatory cytokines and chemokine were shown in a dose-dependent manner in 2–50 ng/ml of IL-33 (Fig. 2B). These stimulatory responses were confirmed at the protein levels with 2-5-fold increase in TNF-α, IL-1β, IL-6 or IL-8 in the culture media from HCECs treated with IL-33 in 2–50 ng/ml (Fig. 2C).

### IL-33 Stimulated Inflammatory Mediators via ST2 Signaling in HCECs

**Figure 3:**
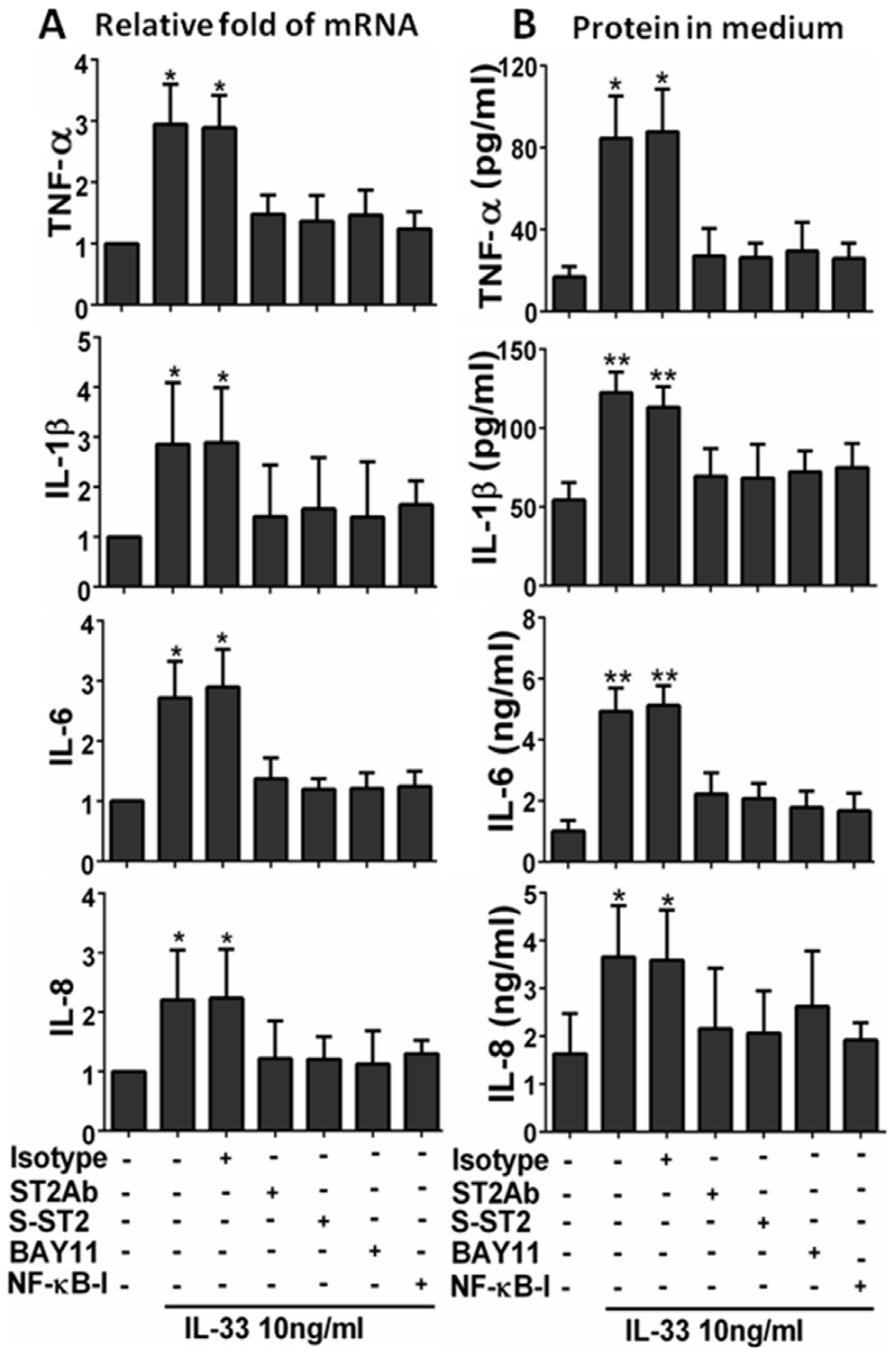
ST2 and NF-κB signaling pathways were involved in IL-33 induced inflammatory response. The HCECs were exposed to IL-33 (10 ng/ml) with prior incubation in the absence or presence of isotype IgG (5 µg/ml), ST2Ab (5 µg/ml), Soluble ST2 protein (S-ST2, 10 ng/ml), BAY11-7082 (10 µM) or NF-κB activation inhibitor quinazoline (NF-κB -I, 10 µM) for 1 h. The cultures treated by IL-33 for 4 h were subjected to RT-qPCR to measure mRNA (**A**), the cultures treated for 48 h were used to evaluate protein in medium supernatants by ELISA (**B**). Results shown are the mean±SD of four independent experiments. *P<0.05; **P<0.01, n = 4.

The role of ST2 in mucosal epithelium is largely unknown. Here we investigated whether ST2 signaling was essential in IL-33-stimulated production of inflammatory mediators by primary HCECs. When treated with 10 ng/ml of IL-33 for 4–48 hours, the production of TNF-α, IL-1β, IL-6, and IL-8 was significantly increased at both mRNA (Fig. 3A) and protein (Fig. 3B) levels. Pre-treatment with 5 µg/ml ST2 antibody or 10 ng/ml of soluble recombinant human ST2 protein one hour prior IL-33, significantly suppressed the expression of TNF-α, IL-1β, IL-6, and IL-8 induced by 10 ng/ml of IL-33 at both mRNA (Fig. 3A, all p<0.05, n = 4) and protein (Fig. 3B, p<0.05 or 0.01, n = 4) levels. These findings suggest that ST2 plays an important role in IL-33 induces inflammation in HCECs.

### IL-33/ST2 Signaling Mediated Inflammatory Responses via NF-κB Pathway in HCECs

**Figure 4:**
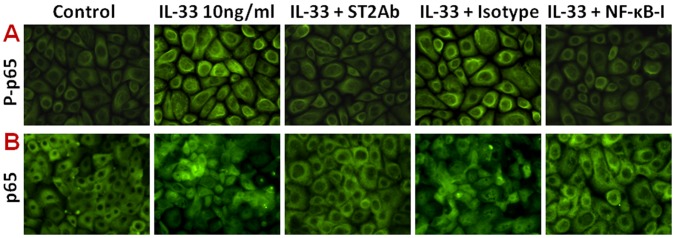
NF-κB activation was induced by IL-33 and inhibited by ST2 antibody and NF-κB activation inhibitor quinazoline (NF-κB-I) in HCECs. The HCECs were exposed to IL-33 (10 ng/ml) with prior incubation in the absence or presence of ST2Ab (5 µg/ml), isotype IgG (5 µg/ml) or NF-κB-I (10 µM) for 1 h. The cells treated by IL-33 for 1 or 4 h in 8-chamber slides were used for immunofluorescent staining with rabbit antibody against phosphor-p65 (P-p65, **A**) or total p65 (**B**), respectively. The representative images were from three independent experiments. Magnifications 400X.

We further investigated whether NF-κB signaling pathway is involved in IL-33/ST2 stimulated inflammatory response in HCECs. As shown in Fig. 3A&B, pre-treatment of NF-κB activation inhibitor quinazoline (NF-κB-I, 10 µM) or IκB-α inhibitor BAY11-7082 (10 µM) significantly suppressed the mRNA expression and protein production of TNF-α, IL-1β, IL-6 and IL-8, stimulated by 10 ng/ml of IL-33 in HCECs (Fig. 3A&B). The NF-κB activation was further confirmed by immunofluorescent staining showing the increased phosphorylation (Fig. 4A) and nuclear translocation (Fig. 4B) of NF-κB p65 protein in HCECs exposed to 10 ng/ml of IL-33. Interestingly, the IL-33 stimulated p65 activation (phosphorylation and nuclear translocation) was markedly blocked by ST2 antibody (ST2Ab, 5 µg/ml), but not by its isotype IgG. Furthermore, p65 activation was also blocked by NF-κB-I (Fig. 4). These results suggest that IL-33 induces inflammatory responses by HCECs via ST2 signaling and NF-κB activation.

## Discussion

A novel proallergic cytokine IL-33, mainly produced by epithelial cells, has been identified as a natural ligand of the IL-1 receptor family member ST2, which is preferentially expressed by Th2 cells and is involved in allergic inflammation [1]. By binding to ST2 receptor, IL-33 can activate Th2 cells and mast cells to secrete Th2 cell-associated cytokines and chemokines that lead to severe pathological changes in mucosal organs [20]. Recent studies showed that the IL-33 and its receptor ST2 play important roles in allergic conjunctivitis [10]. However, the role of IL-33 and ST2 in pro-inflammatory response by ocular surface epithelium has not been well investigated. It is not clear whether ST2 is expressed by corneal epithelial cells. Using fresh donor corneal tissues and primary human corneal epithelial cells, the present study revealed for the first time that ST2 is expressed by normal corneal epithelium; and IL-33/ST2 signaling promotes corneal epithelial cells to secrete inflammatory cytokines and chemokines through NF-κB signaling pathways.

### Human Corneal Epithelial Cells Possess ST2, an IL-33 Receptor

ST2, a member of IL-1 receptor family, has been characterized as a biomarker of inflammatory processes, such as bronchial asthma, idiopathic pulmonary fibrosis, heart failure, dengue virus infection, and septic shock and trauma. ST2 is preferentially expressed by Th2 cells [21], mast cells [22], eosinophils [23], basophils [24] and endothelial cells [25]. Recent studies showed that ST2 was also expressed by epithelial cells [26]. Moreover, soluble ST2 has been recognized as a decoy receptor that competitively binds to IL-33 [27]. Soluble ST2 may confer protection to arthritis [28] and atherosclerosis development [29], heart surgery [30], allergic airway inflammation [31], and warm hepatic ischemia/reperfusion injury [32]. High ST2 serum levels detected in chronic inflammatory processes suggest that soluble ST2 might be involved in controlling the progress of the disease [33]. However, the location and function of ST2 in corneal epithelial still remains unknown.

Our findings showed that the ST2 mRNA was expressed by primary HCECs, and it was stimulated when the cells exposed to IL-33 in a dose-dependent manner (Fig. 1C). As shown in Fig. 1B, the ST2 protein levels significantly increased in HCECs exposed to IL-33, when compared with the untreated control. In ex vivo donor corneal epithelium, ST2 protein was detected to be located in superficial layers, and it was stimulated to multiple layers of the epithelium exposed to IL-33. The findings demonstrate that ST2 is present in human corneal epithelial cells, suggesting a potential role in ocular surface inflammatory diseases.

### IL-33 Mediated Inflammatory Responses via ST2 in HCECs

It is well known that IL-33 is a novel proallergic cytokine involved in allergic inflammation. IL-33 signals through its receptor ST2 and drives production of inflammatory cytokines and chemokines in target cells, including Th2 lymphocytes [34], mast cells [35], basophils and eosinophils [36], endothelial cells [37], and invariant NKT and NK cells [4], [38]. The IL-33/ST2 axis appears to play an important role in several chronic inflammatory disorders, including asthma [8], rheumatoid, arthritis [16], [39], and anaphylactic shock [40]. IL-33/ST2 signaling affects immune response to bacteria and viruses [41], [42]. However, the function of IL-33/ST2 axis in regulating inflammation of ocular surface epithelium has not been reported.

In the present study, we showed that IL-33 significantly stimulated production of proinflammatory cytokines (TNF-α, IL-1β, and IL-6) and chemokine IL-8 by HCECs at both mRNA and protein levels, in a concentration-dependent manner (Fig. 2). Interestingly, the IL-33 stimulated expression and production of these inflammatory mediators were markedly blocked by a ST2 neutralizing antibody or soluble ST2 protein in cultured primary HCECs (Fig. 3). These findings demonstrated that the stimulation of the inflammatory cytokines and chemokine by IL-33 is through activation of its receptor ST2 signaling. Further studies are necessary to clarify the underlying mechanism by which IL-33/ST2 play an important role in inflammatory disease.

### IL-33/ST2 Signaling Mediated Inflammatory Responses via NF-κB Activation in HCECs

NF-κB signaling pathway appears to mediate mucosal epithelial inflammation [43], [44]. We investigated the potential signaling pathway by which IL-33 and its receptor ST2 exert the role in inflammatory response. NF-κB is present in the cytoplasm of resting cells as a dimer bound to an inhibitor protein IκB to form an inactive protein complex. Thus, NF-κB biological activity is controlled mainly by the IkB-α and IkB-β proteins, which restrict NF-κB to the cytoplasm and inhibit its DNA binding activity. Phosphorylation of IκB, which leads to its dissociation from NF-κB protein and subsequent degradation, results in the phosphorylation, release and translocation of NF-κB protein from cytoplasm to nucleus, which promotes the expression of relevant inflammatory genes [45].

This study has demonstrated that NF-κB was dramatically activated with p65 protein phosphorylation and nuclear translocation in corneal epithelial cells exposed to IL-33 for 1 or 4 h respectively (Fig. 4). Quinazoline, a NF-κB activation inhibitor, blocked the NF-κB p65 phosphorylation and nuclear translocation. Interestingly, the stimulated induction of inflammatory cytokines (TNF-α, IL-1β, and IL-6) and chemokine IL-8 by IL-33 were also markedly blocked by Quinazoline and IkB-α inhibitor BAY11. These findings confirmed that IL-33 promotes production of proinflammatory mediators is mediated by the NF-κB signaling pathways.

In conclusion, our findings demonstrate for the first time that ST2 is present in human corneal epithelial cells, and IL-33 stimulates expression and production of proinflammatory cytokines and chemokines through ST2 and NF-κB signaling pathways in corneal epithelial cells. This study suggests that IL-33/ST2 axis may play an important role in ocular surface inflammatory diseases. We may speculate a possible dual effect of ST2 in different target tissues: promoting allergic inflammation through Th2 cells while regulating pro-inflammatory response in epithelial cells.

## Materials and Methods

### Materials and Reagents

Cell culture plates, centrifuge tubes and other plastic ware were purchased from Becton Dickinson (Lincoln Park, NJ). Dulbecco modified Eagle medium (DMEM), Ham F-12, amphotericin B, gentamicin and 0.25% trypsin/0.03% EDTA solution were from Invitrogen GIBCO BRL (Grand Island, NY). Fetal bovine serum (FBS) was from Hyclone (Logan, UT). Primary antibody against ST2 came from Santa Cruz Biotechnology (Santa Cruz, CA). Fluorescein Alexa Fluor 488 conjugated second antibodies (goat anti-mouse or anti-rabbit IgG) were from Molecular Probes (Eugene, OR). Soluble recombinant human ST2 was from Abbiotec (San Diego, CA). Hydrocortisone, human EGF, cholera toxin A subunit, dimethyl sulfoxide (DMSO), Hoechst 33342 and other reagents came from Sigma (St Louis, MO). Affinity purified rabbit polyclonal antibody against p65 was from Santa Cruz Biotechnology (Santa Cruz, CA). A rabbit antibody against phospho-NF-κB p65 was from Cell Signaling Technology (Danvers, MA). ELISA DuoSet kits for human IL-6, IL-8, IL-1β and TNF-α were from BioLegend (San Diego, CA). RNeasy Plus Mini RNA extraction kit was from Qiagen (Valencia, CA). Enhanced Chemiluminescence (ECL) reagents and Ready-To-Go-Primer First-Strand Beads were obtained from GE Healthcare (Piscataway, NJ); TaqMan gene expression assays and real-time PCR master mix were from Applied Biosystems (Foster City, CA).

### Human Corneal Epithelial Tissue ex vivo Model for Inflammatory Cytokine Induction

A fresh corneoscleral tissue was cut into four equal-sized pieces. Each quarter of the corneoscleral tissue was placed into a well of an eight-chamber slide with epithelial side up in 150 µl of serum-free SHEM [46], without or with IL-33 (10 ng/ml) for 24 h in a 37°C incubator. The corneal epithelial tissues were prepared for frozen sections for ST2 immunohistochemical staining.

### Primary Human Corneal Epithelial Culture Model for Inflammatory Cytokine Induction

Fresh human corneoscleral tissues from donors aged 19–67 years were obtained from the Lions Eye Bank of Texas (Houston, TX). Human corneal epithelial cells (HCECs) were cultured in 12-well plates with explants of corneal limbal rims in a supplemented hormonal epidermal medium (SHEM) containing 5% FBS according to our previously reported method [47]. Corneal epithelial cell growth was carefully monitored, and only the epithelial cultures without visible fibroblast contamination were used for this study. Confluent corneal epithelial cultures were switched to serum-free SHEM and treated with IL-33 in different concentrations. Each experiment was repeated at least three times. The cells treated for 1–24 h were lysed for total RNA extraction and evaluating mRNA expression. The supernatants of the conditioned medium and the cell lysate in the cultures treated for 24–48 h were collected and stored at −80°C for immunoassay.

### IL-33/ST2/NF-κB Signaling Pathway Evaluation

HCECs were preincubated with specific ST2 antibodies(5 µg/ml), soluble recombinant human ST2 (10 ng/ml) or pathway inhibitors, BAY11-7082 (10 µM) or NF-κB activation inhibitor (quinazoline 10 µM) for 1 h before IL-33 was added for 1, 4, 6, 24 and 48 hours, respectively [48]. The cells treated with IL-33 for 1 or 4 hours in eight-chamber slides were fixed for immunofluorescent staining to detect NF-κB p65 phosphorylation and nuclear translocation respectively. The cells in 12-well plates were subjected to total RNA extraction for measuring inflammatory cytokine (TNF-α, IL-1β, and IL-6) and chemokine IL-8 expression by RT and real-time PCR. The cultured cells treated for 24–48 h were lysed in RIPA buffer for ELISA.

### Total RNA Extraction, Reverse Transcription (RT) and Quantitative Real-time PCR

Total RNA was isolated from cells using a Qiagen RNeasy® Mini kit according to the manufacturer’s protocol, and quantified by a NanoDrop® ND-1000 Spectrophotometer and stored at −80°C. The first strand cDNA was synthesized by RT from 1 µg of total RNA using Ready-To-Go You-Prime First-Strand Beads as previously described [49]. The real-time PCR was performed in a Mx3005PTM system (Stratagene) with 20 µl reaction volume containing 5 µl of cDNA, 1 µl of TaqMan® Gene Expression Assay for IL-6, IL-8, IL-1β, TNF-α (TaqMan Assay Hs00174131_m1, Hs00174103_m1, Hs01555413_m1, Hs00174128_ m1,) or GAPDH (Hs99999905 m1) and 10 µl Master Mix. The thermocycler parameters were 50°C for 2 min, 95°C for 10 min, followed by 40 cycles of 95°C for 15 s and 60°C for 1 min. A non-template control was included to evaluate DNA contamination. The results were analyzed by the comparative threshold cycle (CT) method and normalized by GAPDH [50].

### Enzyme-linked Immunosorbent Assay

Double-sandwich ELISA for human IL-6, IL-8, IL-1β, TNF-α was performed, according to the manufacturer’s protocol, to determine the concentration of IL-6, IL-8, IL-1β, TNF-α protein in conditioned media and culture cell lysates from different treatments. Absorbance was read at 450 nm with a reference wavelength of 570 nm by a VERSAmax microplate reader (Molecular Devices, Sunnyvale, CA).

### Immunohistochemical and Immunofluorescent Staining

Indirect immunostaining was performed according to our previously reported methods [47]. In brief, human corneal frozen sections or corneal epithelial cells on eight chamber slides were fixed in acetone at −30°C for 5 min. Cell cultures were permeabilized with 0.2% Triton X-100 in PBS at room temperature for 10 min. Primary goat antibody against human ST2 (1∶100, 2 µg/ml ) or rabbit antibody against human p65 (1∶100, 2 µg/ml) was applied for 1 h. A donkey anti-goat biotinylated secondary antibody (R&D Systems) and an ABC peroxidase system (Vectastain; Vector Laboratories, Burlingame, CA) were then used for histochemical staining. For fluorescent staining, Alexa Fluor 488-conjugated secondary antibody was applied for 1 h followed by propidium iodide (PI, 2 µg/ml) for 5 min for nuclear counterstaining. Secondary antibody alone or isotype IgG was used as the negative controls. The results were photographed with an epifluorescence microscope (Eclipse 400; Nikon, Garden City, NY) using a digital camera (DMX 1200; Nikon).

### Statistical Analysis

Student’s t-test was used to compare differences between two groups. One-way ANOVA test was used to make comparisons among three or more groups, and the Dunnett’s post hoc test was used to identify between group differences. P value <0.05 was considered statistically significant.
